# Current Research Status of Fusarium Crown and Root Rot Diseases in Wheat-Growing Countries of North Africa: A Review

**DOI:** 10.3390/pathogens15010069

**Published:** 2026-01-09

**Authors:** Yassine Tanane, Fatiha Bentata, Abderrakib Zahid, Muamar Al-Jaboobi, Rachid Moussadek, Seid Ahmed Kemal

**Affiliations:** 1 Plant Protection Unit, Department of Plant Production, Protection and Biotechnology, Hassan II Institute of Agronomy and Veterinary Medicine, Rabat 10101, Morocco; a.zahid@iav.ac.ma; 2 Research Unit of Plant Breeding and Conservation of Plant Genetic Resources, National Institute for Agricultural Research, Rabat 10101, Morocco; fatiha.bentata@inra.ma (F.B.); r.moussadek@cgiar.org (R.M.); 3 International Center for Agricultural Research in the Dry Areas (ICARDA), Biodiversity and Integrated Gene Management, Rabat 10101, Morocco; m.aljaboobi@cgiar.org (M.A.-J.); s.a.kemal@cgiar.org (S.A.K.)

**Keywords:** Fusarium crown and root rot, wheat, North Africa, cropping systems, disease management, mycotoxins, Brassica

## Abstract

Bread and durum wheat are the most important staple crops, providing 55% of the carbohydrates and 20% of the daily caloric intake for nearly 40% of the global population. However, yield losses in durum wheat can reach up to 56% due to reductions in grain yield and agronomic traits. Local wheat production is increasingly declining because of biotic and abiotic stress. The severity of Fusarium crown and root rot diseases is influenced by cereal mono-culture, specific agronomic practices, and the cultivation of susceptible wheat cultivars. The review highlights current research on the causal agents, economic importance, and management practices of Fusarium crown and root rot diseases in North African countries. The review will contribute to the study of these diseases in wheat.

## 1. Introduction 

Bread and durum wheat (*Triticum aestivum* L. and *Triticum turgidum* L. *durum* Desf.) are the most important staple crops, providing 55% of carbohydrates and 20% of daily caloric intake to nearly 40% of the global population [1]. In North African countries (Morocco, Tunisia, Algeria, and Egypt), wheat is a strategic crop occupying up to 5 million hectares, representing 75% of the total cultivated land [2]. Durum wheat covers approximately 50 to 60% of total cereal areas, particularly in semi-arid and arid zones, and is consumed as couscous, semolina, and traditional breads [3,4,5,6]. Wheat consumption in the North African region provides up to 36% of daily caloric intake and 38% of protein needs, which is higher than the global average [7]. North African countries are wheat import-dependent to meet more than 55% of their wheat consumption [7]. High import dependency makes the countries in the regions vulnerable to global market shocks due to climate change and conflicts. Despite the importance of wheat, its production is constrained by insect pests and foliar- and soil-borne diseases. Among these, Fusarium crown and root rot (FCRR) are important farm binding constraints in wheat-growing dry areas in North Africa.

In this review, we summarize the distribution, importance of FCRR diseases, and their epidemiology and management practices to identify research gaps that need to be addressed by national and international wheat research programs in North Africa. 

## 2. Distribution and Economic Importance of FCRR Diseases of Wheat in North Africa

In North Africa, FCRR diseases are widespread in wheat-growing regions, causing varying levels of yield losses. In Algeria, surveys conducted during 2014 and 2015 cropping seasons recorded high disease severity, reflecting the intensity of symptoms on infected plants, mainly due to drought and heat stress, and the major pathogen isolated was *Fusarium culmorum* [8]. In Morocco, field surveys in different seasons showed that the disease incidence, indicating the proportion of plants affected within a field, ranged from 80 to 93% depending on the wheat species, and the major pathogen identified was *F. culmorum* [9,10,11]. Disease prevalence and incidence were affected by climatic conditions and levels of nitrogen fertilization. In Tunisia, a field survey reported that FCRR incidence was the highest in humid zones (60–100%), followed by sub-humid zones (14–90%) and the lowest was in semi-arid zones (8–22%) [12]. 

Most assessments of yield and agronomic trait losses were derived from disease surveys and a few designed field experiments (Table 1). These types of assessments help to clarify how the losses were measured, since surveys reflect natural field conditions while designed experiments provide more controlled estimates of disease impact. This distinction is important for interpreting variability in yield and agronomic trait losses reported among countries. Yield and agronomic component losses varied across countries depending on weather conditions, crop and disease management practices, wheat growth stages, cultivar susceptibility, and specific pathogens involved in FCRR diseases complex. Most of the studies in the region showed that durum wheat experienced higher yield losses than bread wheat, mainly due to reductions in plant height, grain weight, number of tillers, and overall biomass. The decrease in biomass also contributed to feed shortages in crop–livestock farming systems.

## 3. *Fusarium* Species Causing FCRR Diseases of Wheat in North Africa

Fusarium crown and root rot diseases are mainly caused by different *Fusarium* spp., significantly affecting wheat productivity in North African countries. Isolation from infected roots and crowns of wheat plants revealed multiple *Fusarium* species coexisting in the same fields, all contributing to the disease in the five countries covered in this review. These results indicate that FCRR is caused by a complex of interacting *Fusarium* species rather than a single pathogen (Table 2). The most isolated *Fusarium* species across the North African countries were *F. culmorum*, *F. pseudograminearum*, *F. graminearum*, and *F. oxysporum*. This dominance pattern likely reflects ecological adaptation across the region: *F. culmorum* is consistently the dominant species in the semi-arid wheat-growing areas of North Africa and seems well-adapted to survive on cereal residues under low-moisture conditions, whereas *F. graminearum* is more common in the relatively more humid sub-regions. *F. pseudograminearum* and other species occur only sporadically. Some of the *Fusarium* spp. isolated from diseased crown and roots of wheat are known to cause wilt/root rot diseases of temperate legumes which are key components of the wheat rotation system.

Some of the isolated *Fusarium* spp. are known producers of mycotoxins (DON, ZEA, nivalenol, and T-2 toxin), which pose health risks to humans and livestock. In Morocco, Tunisia, and Algeria, mycotoxin contamination has been reported and represents an important food safety concern [17,18].

## 4. Methods of Identification of *Fusarium* Species

Identification of *Fusarium* spp. is usually performed following traditional methods, which involve isolating the pathogens using potato dextrose agar, carnation leaf agar, or peptone sucrose agar, followed by identification using standard taxonomic keys and descriptions [27,28,29,30]. Initial identification is based on colony morphology, pigmentation, growth rate, and microscopic features of macroconidia and microconidia. However, traditional methods should be supported with molecular tools for more accurate species identification to make FCRR disease management more effective on wheat [31,32]. Molecular identification of *Fusarium* species is increasingly employed in many studies. For example, in Morocco, *Fusarium* species were identified using EF-1α gene to assess the distribution of *F. culmorum* and *F. pseudograminearum* across surveyed wheat farms in different regions [33]. In Tunisia, *Tri5*, *Tri3*, and *Tri7* genes were used to identify trichothecene-producing *Fusarium* spp. [34] and TEF1 gene was used as a molecular marker for species identification and genetic diversity analyses of *F. culmorum* [31]. In Algeria, mating type markers were employed to assess the genetic diversity of *F. culmorum* population [32].

## 5. Symptomatology and Epidemiology of FCRR Diseases of Wheat

### 5.1. Symptomatology

**Figure 1:**
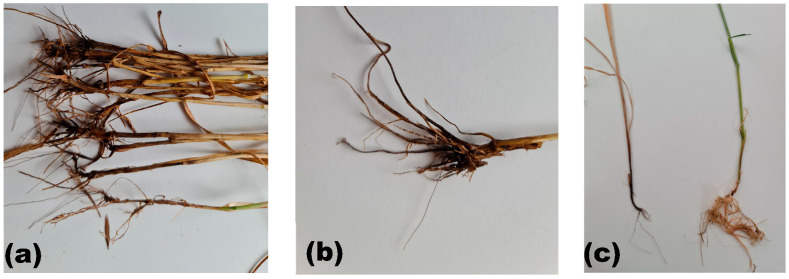
Symptoms of Fusarium crown and root rot (FCRR) in adult wheat plants. (**a**,**b**) Typical symptoms showing dark brown lesions and necrosis on the crown and roots of infected plants; (**c**) comparison between infected (left) and healthy (right) plants. Source: Original figure prepared by the authors.

**Figure 2:**
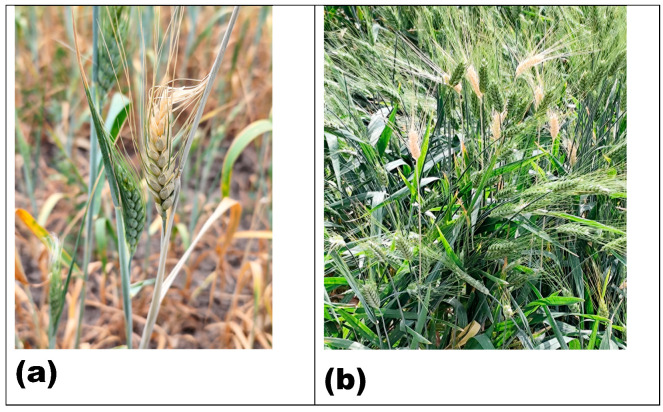
Premature bleaching of wheat heads (“whiteheads”) caused by Fusarium crown and root rot during the grain-filling period (Zadoks 75–85): (**a**) individual whitehead at the plant scale; (**b**) multiple whiteheads observed within an affected field. Source: Original figure prepared by the authors.

Fusarium crown and root rot are major soil-borne disease complexes affecting wheat, particularly in arid and semi-arid regions of North Africa. Effective management of FCRR in these regions requires a thorough understanding of its symptoms and epidemiology in relation to pathogen diversity, genetic variability, survival strategies, cropping practices, and climate variability. Different *Fusarium* spp. affect wheat at different stages of the crop and cause different types of infections. Seedling infections cause blight from seed-borne inoculum [35,36]. Infections on the crown and basal stem cause brown-to-reddish discoloration of the crown and stem base, often accompanied by cell wall reinforcement through lignification as a host defense response, although this response may not completely stop disease progression [37,38]. Root infection results in brown or reddish-brown lesions along the root’s axis caused by the same pathogens that affect crown and stem base. Infection root necrosis, honey-brown discoloration at the tiller base, whiteheads, reduced tillering, and early senescence are experienced, as well as the development of whiteheads, a symptom closely linked to the timing of infection due to disruption of vascular tissues and reduced water transport to developing spikes, ultimately leading to spike bleaching. Whiteheads represent the premature bleaching and senescence of wheat spikes during the transition from milk to dough growth stage. Although whitehead heads are considered a characteristic symptom of FCRR, similar symptoms can be caused by severe under late-season drought stress or with other pathogens [14,39,40,41,42] (Figure 1 and Figure 2). 

### 5.2. Drivers of FCRR Epidemics in North Africa

Several factors, including environmental conditions, pathogen biology, and crop management, play key roles in the distribution and severity of FCRR. The disease is widespread in wheat-growing regions of North Africa, where drought, heat, and agronomic practices contribute to its development [8,11,12].

#### 5.2.1. Drought

Wheat is largely produced as a rainfed crop in many North African countries, where low and irregular rainfall frequently exposes crops to drought stress. Drought is the main obstacle to agricultural productivity in the Central and West Asia and North Africa (CWANA) region, leading to substantial reduction in wheat yields due to terminal water stress, which favors FCRR epidemics [43]. 

#### 5.2.2. Agronomic Practices

Farmers use several traditional and emerging agronomic practices that affect FCRR severity on wheat crops. In Morocco, high inorganic nitrogen fertilization (150–200 kg/ha) increased FCRR severity and the incidence of whitehead symptoms [44]. In addition to the amount of nitrogen applied, the type and timing of application also contributed to increased FCRR severity [45].

Assessments comparing direct drilling with conventional drilling showed that the former increased the average percentage of whiteheads of durum wheat (73%) in Tunisia [46]. In Morocco, a high incidence of FCRR was also associated with high nitrogen fertilization [11]. Monocropping is becoming more common in North African countries, causing declines in productivity and increased disease pressure which pose challenges for farmers [47,48]. Wheat–legume and wheat–cereal rotations are widely practiced in North African countries and significantly influence FCRR incidence and severity on wheat. Studies in Tunisia showed that wheat–barley rotation increased *F. pseudograminearum* infections on durum wheat compared to rotations with chickpea, faba bean, or fallow [49].

#### 5.2.3. Pathogen Survival and Diversity

Pathogen survival and diversity are key factors affecting the effective management of FCRR diseases in wheat production. *Fusarium* species associated with FCRR show diverse survival strategies and genetic and ecological diversity, which explains their coexistence and differing behavior in the field. Limited studies were conducted on pathogen survival and the diversity of *Fusarium* spp. complex. In Algeria and Tunisia, *Fusarium* spp. were isolated from infected crop residues that serve as a primary inoculum source [8,12]. Pathogens such as *F. culmorum* can survive for many years in the soil as chlamydospores [14] while *F. pseudograminearum* persists mainly as mycelium on undecomposed wheat residues [50,51]. This long-term survival reduces the effectiveness of short-term crop rotation and zero tillage [52]. 

Studies have examined the genetic diversity of *F. culmorum* (in Morocco, Tunisia, and Algeria) and *F. graminearum* using RAPD, SSR, ISSR, TEF-1α gene sequencing, and protein profiling. The genetic diversity of the *F. culmorum* population showed limited regional differentiation in each country [31,32,53]. Moreover, pathogen populations showed variations in mycotoxin production and aggressiveness in Algeria and Tunisia [31,54], which was evaluated through chromatographic quantification of trichothecenes and disease severity scoring after artificial inoculation, respectively. In Egypt, distinct genetic groups were identified from *F. graminearum* populations collected in Assiut Governorate [55].

The mating types of *F. culmoroum* and *F. graminearum* were analyzed using mating specific primers and both MAT1 and MAT2 were detected. This indicates a potential for sexual reproduction, which could increase pathogen diversity and contribute to the formation of primary inoculum through airborne ascospores [31,32,34].

## 6. FCRR Disease Management Strategies in North Africa

Managing FCRR is challenging due to the complexity of the disease and the long survival of the pathogen in soil and crop residues. Based on the epidemiological drivers discussed above, several crop management practices can be used to reduce disease pressure. These include growing resistant varieties, seed treatment, crop rotation, using healthy seeds, soil fertility management, irrigation, and adjusting sowing time and planting techniques to minimize conditions that favor disease development. 

### 6.1. Cultural Practices

In North Africa, several studies showed that crop rotation, tillage practices, and nitrogen fertilization reduced disease pressure and maintained wheat productivity [56]. In Tunisia, research demonstrated that no-tillage combined with supplemental irrigation and organic fertilization significantly reduced FCRR severity in durum wheat [14].

Crop rotation with legumes and barley helps reduce FCRR incidence by lowering the buildup of soil-borne Fusarium inoculum, even though these crops are not strict non-hosts and may still allow limited saprophytic survival on their residues. The introduction of Brassica crops in wheat-based rotations in some North African countries, as alternative to drought-tolerant crops [57,58], can reduce pathogen inoculum through bio-fumigation.

Limited research has been conducted on the role of bio-fumigation in controlling FCRR in North Africa. Bio-fumigation is a natural soil disinfestation process in which Brassica plant residues release volatile compounds, particularly isothiocyanates, which have strong antifungal activity against soil-borne pathogens. In a greenhouse experiment, the incorporation of rocket (*Eruca sativa*) and turnip (*Brassica rapa*) residues significantly lowered the severity and incidence of *F. nygamai* infections on bread wheat cultivars [23]. Similarly, Brassica crops (cabbage, turnip, rocket, and radish) were found to mitigate the severity of FCRR and Fusarium head blight caused by *F. pseudograminearum* on wheat varieties under Egyptian conditions [59]. 

In Egypt, field removal of crop residue limited sources of primary inoculum on bread wheat [60]. Deep plowing and improved drainage reduced pathogen buildup in the wheat rhizospheres [61]. 

### 6.2. Conventional and Nano-Based Seed Treatments

Fungicides seed treatments are widely used to control seed and soil-borne pathogens of wheat [18,61,62]. Their primary goal is to reduce the primary inoculum level and protect the roots. Several fungicides are used to treat wheat seeds against the FCRR disease complex. The major products include Metalaxyl-M + Fludioxonil, Triticonazole, Carbendazim, Carboxin + Thiram, Difenoconazole, Azoxystrobin + Difenoconazole and Penconazole in Egypt; and Thiram in Morocco [17,60,61]. Recently, nano-based seed treatments have been emerging as innovative tools to improve the control of seed-borne infections and enhance bioherbicide performance [62,63]. In Egypt, the use of silver nanoparticles (AgNPs at 20 mg/L) applied by seed soaking effectively reduced pre-emergence and post-emergence root rot caused by *F. culmorum* [64].

### 6.3. Biological and Botanical Controls

Biological and botanical approaches play key roles in the development and implementation of biorational strategies for FCRR disease management. In North Africa, most biological and botanical products are applied as seed coatings, while soil drenches are less commonly used. Botanicals refer to plant-derived extracts; however, only limited studies in the region have evaluated essential oils in the management of FCRR in wheat. Most research, conducted primarily under greenhouse conditions and to a lesser extent in the field, have focused on evaluating effective biological control methods focused on antagonistic (*Trichoderma* spp.) and endophytic fungi (*F. subglutinans* and *Meyerozyma guilliermondii*), beneficial bacteria (*Bacillus subtilis*, *Paenibacillus, Pseudomonas* spp.), and actinobacteria (*Streptosporangium becharense*), all of which resulted in good levels of disease reduction caused by *F. culmorum* mainly under greenhouse conditions. Across these studies, microbial agents were most commonly applied as seed treatments, either through seed soaking or seed coating, while some experiments also included soil applications depending on the organism and the experimental design [6,17,65,66,67,68,69,70,71,72]. The mechanisms of actions of these biocontrol agents include direct antagonism, production of siderophores, antifungal compounds, and strong rhizosphere colonization. 

Limited studies were conducted on the use of botanical seed treatments to control FCRR diseases of wheat in North Africa. Plant extracts from *Origanum onites*, *Thymus satureioides*, *Portulaca oleracea*, and *Beta vulgaris* have shown effectiveness in reducing FCRR caused by *F. culmorum.* The essential oils evaluated in the reviewed studies were mainly rich in carvacrol, p-cymene, and thymol, which are compounds typical of *Origanum* and *Thymus* species. Other botanical extracts were generally assessed at the whole-extract level, with their antifungal effects reported without detailed compositional analysis [73,74,75].

### 6.4. FCRR Resistance Breeding

Genetic control involves the development and use of resistant or tolerant wheat cultivars capable of limiting infection or disease severity. Wheat breeding for resistance is one of the most sustainable and effective strategies to prevent FCRR diseases in North Africa. Both national and international (ICARDA and CIMMYT) wheat breeding programs are investing resources in developing elite germplasm and varieties with resistance or partial resistance to FCRR diseases. In Egypt, key bread wheat varieties (Sids-1, Sakha-69, Gimaza-9 and Masr-1) were found susceptible to FCRR diseases caused by *F. nivale*, *F. graminearum*, and *F. tricinctum* [22,24,68,76].

In Tunisia, the widely cultivated durum wheat cv. Karim was found highly susceptible whereas cv. Om Rabiaa showed strong resistance [5,14,77] to FCRR diseases caused by *F. culmorum*. Local durum wheat landraces were more susceptible to FCRR than bread wheat [6,15,44]. In Morocco, researchers identified durum and bread wheat genotypes resistant to FCRR diseases [9,19,45,74]. 

In Morocco, the evaluation of commercial varieties and ICARDA elite durum lines for susceptibility *F. culmorum* infections showed that cv. Marouan was resistant while five ICARDA elite genotypes showed moderately resistant reactions [73]. The national and ICARDA durum wheat breeding program have developed and released cv. Faraj (PI 699898) with good levels of resistance to insects and Fusarium root diseases [78]. In Algeria, landraces of durum and bread wheat showed better resistance to FCRR caused by *F. culmorum* and *F. pseudograminearum* [54,66] and durum wheat to *F. equiseti* [25]. These findings highlight the potential of exploiting local landraces as sources of resistance for incorporation into high-yielding wheat varieties. Within the CIMMYT durum wheat breeding program, genotypes resistant to *F. pseudograminearum* at seedling and adult plant stages (84 and # 197, CIMMYT genotype numbers 7409071 and 7410562) have been identified [79]. Resistant breeding lines developed by ICARDA and CIMMYT can be shared with the national breeding program in North Africa to support regional improvement [80]. 

### 6.5. Integrated Management of FCRR Diseases

Bio-rational disease management, defined as the use of ecologically based, low-toxicity strategies such as biocontrol agents, botanicals, resistant varieties, and cultural practices, is increasingly recognized as a viable approach for managing diseases caused by pathogen complexes. The approach combines multiple complementary strategies to reach synergistic effects and promotes sustainable and resilient disease management in wheat-based production systems of North Africa [81]. In North African countries, integration of control options (fungicides, healthy seeds, seed treatments; biocontrol agents, agronomic practices, and resistant varieties) were reported to effectively manage FCRR diseases caused by different *Fusarium* spp. under greenhouse and field conditions [14,17,60,61,69]. 

### 6.6. Conclusion and Research Gaps in FCRR Diseases in North Africa 

Wheat is a key staple crop in North Africa, and protecting its yield from diseases worsened by drought, monoculture, and poor management is a top priority. Both survey and designed experiments showed that FCRR can cause yield losses that contribute low yield grain and biomass yields in North African countries. Increasing temperatures, recurrent drought, and declining soil moisture strongly favor dryland *Fusarium* spp., particularly *F. culmorum* and *F. pseudograminearum*, increasing the prevalence and severity of FCRR under climate-change scenarios. Drought increases FCRR severity not only by favoring the survival and growth of dryland *Fusarium* spp., but also by weakening wheat’s physiological defenses, making plants more susceptible to infection. Several *Fusarium* spp. have been identified from infected roots and crown, with *F. culmorum* and *F. graminearum* being the dominant pathogens across the region. Shifts in weather patterns and cropping systems may alter the composition of the Fusarium community, potentially increasing mycotoxin risks. Some of the *Fusarium* spp. associated with FCRR are also known to infect temperature food legumes like faba bean, lentil, and chickpea crops, which are vital rotation crops. These crops may act either as alternative hosts or as substrates for saprophytic survival, allowing *Fusarium* spp. to persist in dry environments even in the absence of wheat. Continued monitoring of pathogens associated with FCRR using metagenomics and qPCR will improve our understanding of pathogen dynamics and support breeding programs and management practices. 

Agronomic practices including crop rotation, conservation agriculture, sowing dates, and fertilizer application have shown both positive and negative effects on *Fusarium* spp. diversity and on the incidence of FCRR in the region. It is essential to integrate cultural practices that reduce initial inoculum to decrease FCRR severity and incidence at different growth stages of wheat. Minimizing wheat monoculture through introduction of legumes and Brassica improves soil health and reduces FCRR impacts. Supplementary irrigation also plays a key role in minimizing FCRR by alleviating drought stress on the crop. 

Some Fusarium isolates behave as seed endophytes, remaining latent inside apparently healthy seeds and serving as a primary inoculum source once the crop is sown, which further highlights the importance of seed-targeted biopesticide formulations. Seed-applied biopesticides act by colonizing the seed surface or emerging roots, reducing seedborne Fusarium inoculum and providing early biological protection during the most susceptible seedling stages. The development and deployment of effective biopesticide to control seed-borne and soil-borne *Fusarium* spp. are still in the early stages in the region. Effective biocontrol agents used as consortia together with suitable formulation and delivery system can contribute to reducing the initial inoculum and FCRR diseases in wheat fields.

Taken together, the literature highlights three major areas that are central to understanding and managing FCRR in North Africa. First, the distribution and diversity of *Fusarium* species remain critical to identify the most prevalent pathogens across wheat-growing regions. Second, screening of durum and bread wheat germplasm under greenhouse and field conditions is essential to evaluate levels of resistance and susceptibility. Screening typically measures crown discoloration, root necrosis, seedling vigor, and yield performance under Fusarium inoculation, which together indicate the level of resistance. Third, advances in genomic tools such as genome-wide association studies (GWAS) provide new opportunities to dissect the genetic basis of resistance and to support breeding programs aimed at developing FCRR-resistant wheat varieties. Integrating these complementary approaches offers promising perspectives for sustainable management of FCRR in the region.

In North African countries, the growing conditions and the pathogens associated with FCRR are similar. Therefore, it requires regional research and capacity-building partnerships, bridging the gap between research and application. The partnerships can serve as a platform to strengthen germplasm exchange and ensure innovations meet the needs of farmers, thereby having wide impacts in the region.

## Figures and Tables

**Table 1:** Yield and agronomic component losses due to FCRR from surveys and designed studies in North Africa.

Country	Type of Wheat	Grain Yield Loss (%)	Loss of Agronomic Traits	Type of Study	References
Tunisia	Durum wheat	Up to 44%	Reduced spike number and grain weight	Survey	[13]
		38% (cv. Karim), 17% (cv. Om Rabiaa)	Biomass and grain weight loss	Controlled conditions	[14]
		26%	Shorter plants, fewer tillers	Designed field experiments	[5]
	Bread wheat	<15%	Minor grain weight loss	Designed field experiments	
	Durum wheat	51–56%	Grain weight reduction, spike abortion	Designed field experiments	[15]
	Bread wheat	6–44%	Lower grain weight and spike fertility	Designed field experiments	
Morocco	Unspecified	13.4–14.6%	Reduced plant height, thousand kernel weight	Survey	[11]
		4.9–10.6%	Grain-filling loss, biomass reduction	Survey	
		12–17%	Reduced head length, kernel number	Survey	[16]
		20–51%	Spike sterility, poor tillering	Survey	[9]

**Table 2:** *Fusarium* species associated with FCRR diseases of wheat in North Africa.

Country	Wheat Types Infected	Dominant Pathogens	Other *Fusarium* Species	Potential Mycotoxin-Producing *Fusarium* spp.	References
Morocco	Bread and Durum	*F. culmorum*, *F. graminearum*	*F. oxysporum*, *F. solani*, *F. poae*, *F. equiseti*, *F. sambucinum*, *F. avenaceum*, *F. nivale*, *F. redolens*, *F. moniliforme, F. pseudograminearum*	*F. culmorum*, *F. graminearum*, *F. poae*, *F. avenaceum*, *F. moniliforme*, *F. sambucinum*, *F. nivale, F. pseudograminearum*	[11,16,17,18,19]
Tunisia		*F. culmorum*, *F. pseudograminearum*	*F. oxysporum*, *F. solani*, *F. equiseti*, *F. compactum*, *F. verticillioides*, *F. avenaceum*, *F. anthophilum*, *F. algeriense*, *F. brachygibbosum*, *F. nygamai*, *F. redolens*	*F. culmorum*, *F. pseudograminearum*, *F. verticillioides*, *F. avenaceum*, *F. sambucinum*	[7,13,20,21,22]
Egypt	Bread and Durum	*F. culmorum*, *F. oxysporum*	*F. nygamai*, *F. solani*, *F. moniliforme*, *F. graminearum*, *F. nivale*, *F. tricinctum*	*F. culmorum*, *F. graminearum*, *F. tricinctum*, *F. moniliforme*, *F. nivale*, *F. nygamai*	[22,23,24]
Algeria	Mostly Durum	*F. culmorum*, *F. graminearum*	*F. cerealis*, *F. acuminatum*, *F. oxysporum*, *F. asiaticum*, *F. brachygibbosum*, *F. verticillioides*, *F. avenaceum*, *F. equiseti, Microdochium* spp., *F. incarnatum*	*F. culmorum*, *F. graminearum*, *F. cerealis*, *F. asiaticum*, *F. verticillioides*, *F. avenaceum*	[8,20,21,25,26]

## Data Availability

No new data were created or analyzed in this study. Data sharing is not applicable to this article.

## Abbreviations

The following abbreviations are used in this manuscript:

ICARDA	International Center for Agricultural Research in the Dry Areas
INRA	National Institute for Agricultural Research
FCRR	Fusarium Crown and Root Rot
CGIAR	Consultative Group on International Agricultural Research
DON	Deoxynivalenol
ZEA	Zearalenone
CWANA	Central and West Asia and North Africa
CIMMYT	International Maize and Wheat Improvement Center
qPCR	quantitative Polymerase Chain Reaction
GWAS	Genome-Wide Association Study
